# Retraction Note: The best drug supplement for obesitytreatment: a systematic review and network meta-analysis

**DOI:** 10.1186/s13098-023-01048-3

**Published:** 2023-04-17

**Authors:** Nader Salari, Samira Jafari, Niloofar Darvishi, Elahe Valipour, Masoud Mohammadi, Kamran Mansouri, Shamarina Shohaimi

**Affiliations:** 1 Department of Biostatistics, School of Health, Kermanshah University of Medical Sciences, Kermanshah, Iran; 2 Student Research Committee, Kermanshah University of Medical Sciences, Kermanshah, Iran; 3 Zimagene Medical Genetics Laboratory, Avicenna St, Hamedan, Iran; 4 Department of Nursing, School of Nursing and Midwifery, Kermanshah University of Medical Sciences, Kermanshah, Iran; 5 Medical Biology Research Center, Kermanshah University of Medical Sciences, Kermanshah, Iran; 6 Department of Biology, Faculty of Science, University Putra Malaysia, Serdang, Selangor, Malaysia

Retraction: Salari et al. Diabetol Metab Syndr _#####################_

10.1186/s13098-021-00733-5

The Editor-in-Chief has retracted this article. After publication, concerns were raised regarding the reliability of the reported data, which the authors addressed by a Correction [1]. However, further issues were subsequently identified with the authors potentially reporting mean differences as standardized mean differences. Post-publication peer review has concluded that the authors’ methodology contains technical and statistical errors, which undermine the conclusions of the article.

Authors Kamran Mansouri and Masoud Mohammadi disagree with this retraction. The other authors have not responded to correspondence regarding this retraction.

## References

[1] Salari, N., Jafari, S., Darvishi, N. et al. Correction to: The best drug supplement for obesity treatment: a systematic review and network meta-analysis. Diabetol Metab Syndr 14, 68 (2022). 10.1186/s13098-022-00838-5 10.1186/s13098-022-00838-5 PMC9077956 35526058

